# The costs of introducing artemisinin-based combination therapy: evidence from district-wide implementation in rural Tanzania

**DOI:** 10.1186/1475-2875-7-4

**Published:** 2008-01-07

**Authors:** Joseph D Njau, Catherine A Goodman, S Patrick Kachur, Jo Mulligan, John S Munkondya, Naiman Mchomvu, Salim Abdulla, Peter Bloland, Anne Mills

**Affiliations:** 1 Ifakara Health Research & Development Centre, PO Box 78373, Dar es Salaam, Tanzania; 2 Health Policy Unit, London School of Hygiene & Tropical Medicine, Keppel St., London, WC1E 7HT, UK; 3 KEMRI/Wellcome Trust Collaborative Programme, PO Box 43640, Nairobi, Kenya; 4 Division of Parasitic Diseases, United States Centers for Disease Control and Prevention, 1600 Clifton Road, Mailstop F-22, Atlanta, GA 30333, USA

## Abstract

**Background:**

The development of antimalarial drug resistance has led to increasing calls for the introduction of artemisinin-based combination therapy (ACT). However, little evidence is available on the full costs associated with changing national malaria treatment policy. This paper presents findings on the actual drug and non-drug costs associated with deploying ACT in one district in Tanzania, and uses these data to estimate the nationwide costs of implementation in a setting where identification of malaria cases is primarily dependant on clinical diagnosis.

**Methods:**

Detailed data were collected over a three year period on the financial costs of providing ACT in Rufiji District as part of a large scale effectiveness evaluation, including costs of drugs, distribution, training, treatment guidelines and other information, education and communication (IEC) materials and publicity. The district-level costs were scaled up to estimate the costs of nationwide implementation, using four scenarios to extrapolate variable costs.

**Results:**

The total district costs of implementing ACT over the three year period were slightly over one million USD, with drug purchases accounting for 72.8% of this total. The composite (best) estimate of nationwide costs for the first three years of ACT implementation was 48.3 million USD (1.29 USD per capita), which varied between 21 and 67.1 million USD in the sensitivity analysis (2003 USD). In all estimates drug costs constituted the majority of total costs. However, non-drug costs such as IEC materials, drug distribution, communication, and health worker training were also substantial, accounting for 31.4% of overall ACT implementation costs in the best estimate scenario. Annual implementation costs are equivalent to 9.5% of Tanzania's recurrent health sector budget, and 28.7% of annual expenditure on medical supplies, implying a 6-fold increase in the national budget for malaria treatment.

**Conclusion:**

The costs of implementing ACT are substantial. Although drug purchases constituted a majority of total costs, non-drug costs were also considerable. It is clear that substantial external resources will be required to facilitate and sustain effective ACT delivery across Tanzania and other malaria-endemic countries.

## Background

For over a decade, malaria treatment in sub-Saharan Africa and other endemic regions has been in crisis. Conventional antimalarial drugs such as chloroquine and sulphadoxine/pyrimethamine (SP) have become increasingly obsolete in the face of growing drug resistance. Current debates favour using artemisinin-based combination therapy (ACT) regimens [1,2]. ACTs are highly efficacious and offer potential to check the progression of drug resistance. They are also expensive with prices as much as 10 to 20 times greater than conventional monotherapies [3,4].

By early 2006, 34 countries in sub-Saharan Africa had adopted ACTs as first-line treatment for malaria [5,6]. The region has the largest number of people exposed to stable malaria transmission and constitutes the greatest burden of malaria morbidity and mortality in the world with pregnant women and children under five years being the most affected groups [7]. Of the 34 countries, only 10 were actively deploying ACT drugs in their public sector [6] and the majority of malaria patients continue to be treated with largely ineffective conventional antimalarial drugs. Despite support from the Global Fund to fight AIDS, Tuberculosis and Malaria, public health specialists remain concerned that many countries in Africa are failing to achieve wide coverage of ACTs. Weak health systems, chronic under funding of public health services and lack of trained and motivated health workers are some of the frequently cited reasons for delays in implementation [4,8]. In addition, the potential benefits of ACT for inhibiting malaria transmission and drug resistance have not been demonstrated in areas of intense malaria transmission and severely constrained health infrastructure – precisely the conditions that prevail in most African countries.

There is relatively little evidence on the actual costs associated with changing national malaria treatment policies to accommodate ACT. Three studies have provided estimates of ACT procurement costs for Tanzania or Africa more generally, but none have included other activities such as training, developing and printing clinical guidelines, behaviour change communication (BCC) and publicity materials, or other supplies [9-11]. Moreover the drug cost estimates in these studies have been obtained from mathematical models rather than records of actual financial costs incurred during implementation in a real world setting. The change in treatment costs at the health facility level as a result of ACT introduction has been estimated from the patient perspective in Senegal [12], and from the provider perspective in South Africa [13], but neither study attempted to document the overall costs of policy implementation. Only one study has assessed the non-drug costs of changing national malaria treatment policy, based on expenditures recorded during the change from chloroquine to SP monotherapy in Tanzania [14]. However, no studies have systematically documented the non-drug costs of introducing more expensive and complex ACT regimens. The current study aims to address this gap.

### Policy context

Tanzania is facing the challenge of increasing antimalarial drug resistance. By the late 1990's chloroquine resistance had reached unacceptable levels, failing to provide an adequate clinical and parasitological cure in more than half of the children studied [15,16]. In 2001, the country took a decision to adopt SP as an interim first-line antimalarial treatment with a clear intention to search for a more lasting solution to the drug resistance problem [17]. As part of this policy change the Tanzanian Ministry of Health prioritized operations research to identify a suitable first-line drug candidate and to gain experience with implementing ACT on a programmatic scale. Among these efforts was the Interdisciplinary Monitoring Project for Antimalarial Combination Therapy in Tanzania (IMPACT-Tz).

IMPACT-Tz was a large-scale study intended to evaluate the implementation of ACT in a typical rural district with intense malaria transmission. It involved a comparison of ACT implementation in Rufiji District (using SP plus artesunate), with continued use of the national first-line drug, SP, in two adjacent districts. IMPACT-Tz was one of the first large scale ACT evaluations in sub-Saharan-Africa under 'real life' conditions in which ACT was delivered through existing public health infrastructures with minimal alterations. It aimed to produce robust results, applicable in similar health resource constrained settings in rural sub-Saharan Africa. The study was explicitly designed to answer key operational research questions including those related to: antimalarial drug resistance, malaria transmission, safety, efficacy, costs and affordability, health seeking behaviour, perceptions and community attitudes towards ACT use.

In 2004, following initial results from the IMPACT-Tz study and other sites participating in the East African Network for Monitoring Antimalarial Treatment [18], the Tanzanian Ministry of Health through the National Malaria Control Programme (NMCP) declared its intention to adopt the ACT artemether-lumefantrine (ARLU) as the national first-line drug to treat uncomplicated malaria. NMCP submitted an ACT implementation grant application to the Global Fund which was awarded later the same year. In 2005, the country began the process of initiating this second treatment policy change, which culminated in the large scale public sector deployment of ARLU in December 2006 [19]. Identification of malaria cases has remained primarily dependant on clinical diagnosis.

This report presents findings from the IMPACT-Tz evaluation on the district-wide costs for three years of activities deploying ACT in Rufiji District. In addition, the projected costs of various ACT policy implementation options at the national level are estimated.

## Methods

### Study site

**Figure 1:**
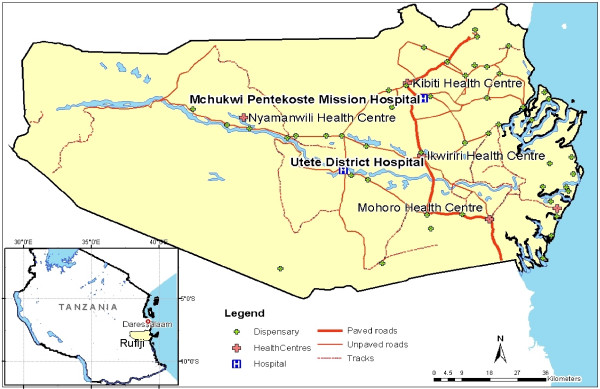
Map of Rufiji District indicating key health facilities.

Rufiji District is located in Coast Region 178 km south of Tanzania's commercial capital, Dar es Salaam. The District covers an area of 13,339 km^2^, with an estimated population of 212,144 [20]. See Figure 1 for detailed location and distribution of health facilities in Rufiji District.

Annual rainfall ranges between 800–1,000 mm in the Rufiji River Basin and malaria transmission occurs all year round, with an entomological inoculation rate of 79–1209 infectious bites per person per year [21]. Malaria is the leading cause of morbidity and mortality across the district [22]. According to routine health records, Rufiji District has one of the highest rates of outpatient consultations for malaria in the country of 826 diagnoses per annum per 1,000 population [23].

The district has a total of 59 health facilities of which 46 are public, 10 mission-run and only three private for profit. Public health facilities are managed by the Council Health Management Team (CHMT) which is based in the district administrative town of Utete. The health facilities are managed through eight administrative blocks called '*cascades*' with between four to nine health facilities per cascade. Non-governmental health care providers are urged to operate within the Ministry of Health and Social Welfare's standard operating procedures, while receiving supervision from the CHMT.

### Description of the intervention

Artesunate (AS) plus SP was introduced for first-line treatment of uncomplicated malaria at all health facilities in Rufiji District from February 2003. This particular combination was introduced not because it was expected to be a future option for first-line treatment, but because it was immediately available, provided an ideal opportunity to gain experience with the ACT approach and could be evaluated against SP monotherapy, the recommended first-line treatment. The design and implementation of the intervention involved a broad spectrum of activities encompassing consultation, building consensus, policy formulation, developing treatment guidelines and other communication materials, training, procurement, repackaging, drug supplies management, and sensitization campaigns. The chronology of these activities is summarized in subsequent Table 1.

**Table 1:** Chronological timeline of ACT program implementation in Rufiji District

**Start date**	**Activity**
Sep-02	Appointment of implementation manager marking the formation of the core project implementation team
Oct – Dec-02	Planning meetings & development of IEC* materials
Nov-02	Rapid assessment of drug requirements, storage, distribution and consumption in Rufiji District
Dec-02 – Jan-03	First and second pre-testing of IEC* materials
Jan-03	First printing of posters and training
Jan-03	First consignment of drugs delivered to Kibiti health centre
Feb-03	Training of health personnel from the 56 district health facilities
Apr-03	Local leakage consultant hired to review drug supplies mechanisms in Rufiji District
Jun-03	Second printing of posters
Jun-03	Second drug consignment delivered to Kibiti health centre
Aug-03	Public Launch of new treatment policy in Rufiji District
Aug-03	Third drug consignment delivered to the 8 cascade supply stations
Sep-03	Fourth drug delivery to the cascades including health facilities in the peripheral areas
Nov-03	Fifth drug delivery
Jan-04	Re-packaging of drugs began
Feb-04	Community meetings
Feb-04	Third printing of posters
Feb-04	Sixth drug delivery
Apr-04	Seventh drug delivery
May-04	Adverse drug reaction training
Jun-04	Strengthening of various measures to mitigate drug leakages
Jun-04	Eighth drug delivery
Sep-04	Ninth drug delivery
Oct-04	Training of school teachers and community leaders
Jan-05	Tenth drug delivery
Feb-05	Refresher training of health personnel from 56 district health facilities
Feb-05	Integration of malaria awareness campaigns into primary school program
May – Jul-05	11^th^, 12^th ^and 13^th ^drug delivery
Aug – Sep-05	Last community sensitization meetings throughout the district

*IEC = Information, education and communication

### Project team recruitment, consultation and planning

A stakeholders' meeting was held in Dar es Salaam in October 2002, and key decisions regarding the implementation process were discussed and agreed [24]. The meeting involved representatives from the research project, the Rufiji CHMT and the NMCP. Also represented were the Medical Stores Department (MSD) – responsible for procurement and distribution of essential medicines – and the Pharmacy Board (which later became the Tanzania Food and Drug Authority). Secondly, a rapid assessment of drug management in the district was carried out by external consultants. Their report documented that drug storage, distribution and the control of drug inventories were inadequate and poorly maintained [25]. Attempts were made to address some of these shortfalls in order to guarantee smooth implementation of the ACT programme. These included new drug stock log-books and improvements in drug distribution mechanisms to all health facilities including the most remote. By November 2002, the core project implementation team included the project implementation manager, one clinician responsible for tracking reported adverse drug reactions, and an administrative assistant. Aside from these three project staff, the rest of the implementation depended on existing personnel within the Council Health Management Team and individual health facilities.

### Preparation of IEC materials and drug package envelopes

In late 2002, an artist worked with the implementation team among communities in Rufiji District to develop communications and dosing instruction illustrations to aid care-seekers and health workers during drug prescription. Five community meetings of between 10 to 30 people were held across the district during the design of these information, education and communication (IEC) materials. Dosing envelopes showing prescription instructions were developed for four age groups: children less than one year, children under-five, school aged children and adults [26]. These were designed to enhance accurate ACT dosing practices by both health workers and patients. In addition, posters and wall charts were developed to explain the ACT and its correct use at the point of dispensing.

### Training

Training of Rufiji District health personnel began in January 2003. The first training involved at least two staff (prescribers and drug dispensers) from all 56 public and mission health facilities. The training was carried out in a centrally located primary school over two days. Training focused on indications and exclusion criteria for the use of ACT, age specific dosing instructions, and the importance of emphasising adherence to dosage regimens. Clinicians were trained to identify severe or cerebral malaria cases and to abide by national treatment guidelines for such cases. The training also included a module on identifying clinical signs of adverse drug reactions resulting from use of prescribed antimalarial drugs. All training activities were conducted by the CHMT with assistance from the IMPACT-Tz project including the principal investigators, some of whom had experience with national level training during the 2001 change of national malaria treatment policy. Following this initial workshop, participants were charged with conducting similar training sessions with health facility staff under their supervision upon returning to their posts. A follow-up training (refresher course) was conducted 18 months after initial ACT introduction, and later that year a separate training course was organized on adverse drug reactions for supervising health workers at all facilities.

Training for community leaders and primary school teachers was conducted two years after ACT introduction to increase awareness in the community and sensitize communities to the health and socio-economic consequences of malaria. Project staff trained supervising clinicians at each of the eight administrative blocks (cascades) who then went on to train 104 primary school teachers and 16 ward community leaders as well as volunteer village health workers.

### Drug procurement and distribution

Between January 2003 and September 2005 over 450,000 adult doses of ACT were deployed to public and mission health facilities in Rufiji District. MSD continued to supply SP in the district, while the project procured artesunate. Because no co-formulated or co-packaged product was available, the drugs were co-administered at the health facility level. Artesunate tablets (Arsumax^® ^50 mg, Sanofi-Aventis, Gentilly, France) were purchased from a single European supplier at an internationally negotiated discount price. The first consignment of artemisinin drugs was delivered to Kibiti Health Centre in January 2003. Kibiti was chosen as a delivery point because all MSD drugs were delivered to this centre, from where they were collected by the cascades and taken to their respective health facilities. For this initial consignment, the district health management team was responsible for ensuring drug distribution to each health facility.

To optimize adherence to recommended dosing regimens, the first ACT dose was directly observed at the health facility. Supplies purchased to facilitate this included vessels for clean water, drinking cups, spoons, knives for cutting tablets, water purifying solution and in some facilities bottles of drinking water. ACT was freely available to all malaria patients seeking care in all public and mission (not for profit) health facilities in Rufiji District.

### Combating drug leakages

In April 2003, following reports that artesunate tablets purchased for the project had been found in shops in Dar es Salaam, an inventory management consultant was hired to work with the project manager. After careful evaluation of the whole drug delivery process, the consultant recommended measures to reduce the risk of future misdirection of medicines. These included the introduction of drug stock audit books for all health facilities receiving project drugs, routine and random audits of drug stock inventories at all levels of the system, and the delivery of artesunate drugs directly to the eight cascades by project vehicles from Dar es Salaam rather than the central point in Kibiti.

Another measure to curb drug leakage involved hiring part time workers in Dar es Salaam to pack the artesunate into large boxes marked with health facility names and the total number of doses the facility was to receive. A 'mystery shopper' also visited shops and private health facilities in Rufiji and Dar es Salaam to identify any vendors illegally stocking project drugs.

### Communication and publicity

In August 2003, a major public launch of ACT was held in Ikwiriri, the largest market center in Rufiji District. This public event was attended by diplomats, politicians and public health officials, journalists and media representatives. The event included community sensitization activities, involving live music, theatre, performing artists and road show vehicles that visited communities across the district. The public launch was followed by a series of community sensitization meetings carried out by the project team in collaboration with the CHMT and community leaders. The last sensitization meeting in August/September 2005 was judged to mark the end of ACT implementation activities in Rufiji District and the start of the transition to ARLU.

### Costing methods: district study

The main objectives of the costing study were to a) document the financial costs of introducing an ACT in a typical malaria endemic district of rural Tanzania, and b) estimate the costs of national implementation. The costing approach was incremental in the sense that it excluded the costs of health facility infrastructure, supervision and staff, which were assumed not to change as a result of the introduction of a new drug. All costs are presented in 2003 prices in Tanzanian shillings and US dollars, based on an exchange rate of 1 USD: TZS 1065 [27]. The 2004 and 2005 prices were deflated to 2003 price values using the annual inflation rates of 4.2 and 4.5 percent, respectively [28].

Costs were calculated from the provider perspective, including only those costs incurred by the public health sector or the project. No Ministry of Health and Social Welfare costs above the district level were included, reflecting the emphasis on decentralization of management functions to districts. Costs to households were excluded, but are not expected to have been significantly different from those incurred under monotherapy.

The timeframe for the costing exercise was defined as the three year period from the initial stakeholders' meeting in October 2002 to the last sensitization meeting held in August/September 2005. This period was selected as it covered all the set up activities needed to implement ACT, and avoided overlap with preparations for nationwide introduction of artemether-lumefantrine, for which initial preparations began in late 2005. Including drug costs for the three year period allowed for year on year variation in malaria prevalence, health facility utilization and drug use to be included, an important element seldom considered when estimating national ACT needs from models alone.

This study presents both ACT programme implementation set-up costs and recurrent costs. Set-up costs were defined to include all activities required to introduce the new policy, which were not repeated regularly. They included costs related to the project implementation team (including local and international consultancy fees), purchase of office equipment (minor capital goods such as computers, vehicles, motorbikes, printers and a mobile radio station), development of IEC materials (including the time of the artist and research team, refreshments for focus group participants etc), and training of health personnel (including transport allowances and per-diems, training venue, refreshments, and training materials etc.). In addition, set-up costs incorporated communication and publicity activities mainly directed to the general public which included road shows, community sensitization meetings, the programme launch and sensitization materials such as stickers, road signs, posters and t-shirts. Most set-up costs were incurred in the first year, with the exception of some additional training courses and community sensitization meetings. Recurrent costs encompassed drug purchase and distribution and other supplies including stationery and office supplies, drug repackaging materials including prescription envelopes, water treatment solution, buckets, scissors, and drug audit books.

The ACT used in the study was not co-formulated. As described above, artesunate was bought separately by the project and co-administered together with SP drugs which were delivered through normal drug delivery channels within the district. SP quantities were collected from drug supply log books across Rufiji District and cross-checked against numbers obtained at the MSD headquarters. The SP drugs were valued at prices provided by MSD, plus 14% transport and distribution charges (standard MSD charging practice for mainland Tanzania).

Cost data were drawn from both expenditure and budget records, routinely documented and maintained by the project accountant who was informed from the start that comprehensive records would be required for the costing exercise. Additional information was obtained through interviewing the IMPACT-Tz project implementer, the accountant, the principal investigators and key district health personnel. Care was taken to exclude research costs, but to include all activities necessary for implementation, including the time of project staff spent on implementation and monitoring activities.

Annualized costs for goods with a lifespan of more than a year were calculated using a 5% discount rate. Vehicles were estimated to have a lifespan of 10 years, motorbikes and the mobile radio base station five years, and computers and printers three years.

### Estimating the costs of national level ACT implementation

The district costs were then extrapolated to estimate the costs of national level implementation for the Tanzanian mainland. It was assumed that all mainland Tanzania would need ACT because the entire country is at risk of malaria [29]. The islands of the Zanzibar Archipelago were excluded because they are governed by an autonomous administration with its own Malaria Control Programme and a distinct antimalarial treatment policy. It was assumed that ACT deployment would follow current public health treatment practices where drugs are largely offered for free in most public health facilities, while the mission facilities continue to offer services on a not-for-profit basis.

Since the Ministry of Health and Social Welfare has adopted ARLU as its nationwide treatment recommendation, all national cost estimates were based on ARLU procurement costs recently announced by the manufacturer (Novartis^® ^Pharma, Basel, Switzerland), ranging from 0.45 USD for children under five to 1.8 USD per adult dose [30]. Unlike SP+AS, ARLU is available in a co-formulated tablet packaged in unit-doses specifically designed for four age or weight groups. However, ensuring these reach each health facility in appropriate ratios will require additional repackaging. It was assumed that this could be accomplished with a level of effort similar to repackaging SP+AS that occurred in the intervention district. The breakdown of malaria outpatient diagnoses by age group was estimated based on IMPACT-Tz 2004 household survey data (Table 2) (IMPACT-Tz unpublished data).

**Table 2:** Parameters used in estimation of nationwide costs and sensitivity analysis

**Calculation Parameters**		
***Rufiji District Parameters***		***Source/Ref. Number***

Total Rufiji District population	212,144	NBS Ref # 20
Malaria outpatient cases per 1000 population in Rufiji District	826	NMCP Ref # 23
Total public health facilities in Rufiji District	46	Rufiji CHMT
Total mission health facilities in Rufiji District	10	
Total ACT (AS + SP) doses deployed over 3 years in Rufiji District	445,944	IMPACT-TZ Annual Reports
***National Parameters for Tanzania Mainland***		
National population growth rate per annum	0.023	NMCP Ref # 23
Total population in Tanzania (2002 census)	33,461,849	
Malaria outpatient cases per 1000 population	390	
Total number of Districts in mainland Tanzania	119	NBS Ref # 20
Total number of public health facilities in mainland Tanzania	3,456	Ministry for Health & Social Welfare, Tanzania
Total number of mission health facilities in mainland Tanzania	951	
MSD handling and distribution costs as proportion of drug costs	0.14	Medical Stores Department
***Drug Cost Parameters***		
Average cost of AS+SP per dose	TZS. 1,870	IMPACT-Tz
Average cost of ARLU per dose	TZS. 1,266	Implementation costs ef # 28
*Proportions of malaria patients by age group:*		
Age between 3 to 36 months	0.34	
Age between 37 to 84 months	0.15	IMPACT-Tz 2004
Age between 7 to 14 years	0.06	household survey
Age from 15 years and above	0.45	
***Sensitivity Analysis***		
Cost of six deputy managers (3 years salary and benefits)	70,986 USD	Ref # 14 (New salary schemes)
Total Expatriate costs	36,672 USD	Ref # 14
Additional office equipments	250,072 USD	IMPACT -Tz. purchase records
Projected increase in public and mission health facility utilization following introduction of ACT	50%	IMPACT-Tz. Surveys
Average cost per dose of cheaper alternative ACT (AS+Amodiaquine)	0.85 USD	1^st ^line drug of choice in Zanzibar

### Scenarios for estimating national costs

For the purpose of estimating national costs, all activities were divided into fixed and variable items (which differed from the earlier categorization of set-up and recurrent). Fixed costs were those that were not expected to vary by scale of implementation, and included the project implementation team, office equipment and the development of IEC materials. Costs for IEC materials and office equipment were based directly on those incurred in Rufiji District. For the costs related to the project team, the approach used in the 2001 study on antimalarial drug policy change from chloroquine to SP was adopted [14], assuming that a similar team (including eight months expatriate time) would be used for nationwide ACT deployment. Personnel costs for the implementation team included 15 months each for the programme and deputy programme manager, the eight months expatriate time, 16 months for a technical officer, 19 months for a supplies assistant, 21 months for a data manager and 84 months of health officer time.

Nationwide variable costs related to drug purchase and distribution, other supplies, training, communication and publicity were estimated by scaling up variable costs incurred in Rufiji District according to four different scenarios, as described below. The one exception was drug distribution costs. As distribution on a nationwide scale would necessarily be undertaken through MSD, distribution costs were based on the MSD standard rate of 14% of drug purchase costs.

#### Scenario I – scaling up by number of districts

Rufiji District variable costs were scaled up by the number of districts in mainland Tanzania (119). This approach assumes that districts are uniform, ignoring variations in population, outpatient malaria diagnoses, number of health facilities etc.

#### Scenario II – scaling up by population

Rufiji District variable costs per capita were scaled up by the total estimated population of mainland Tanzania: 37,491,094 in 2007 (projected from the 2002 national census, at a growth rate of 2.3% [20]). Given the variations in outpatient malaria diagnoses across districts, this may lead to biased results, especially as Rufiji District has a relatively high outpatient malaria diagnosis rate of 826 per 1,000 compared with a national average of 390 per 1,000 [23].

#### Scenario III – scaling up by malaria outpatient diagnoses

Rufiji District variable costs per malaria outpatient diagnosis were scaled up by the total reported malaria outpatient visits for the Tanzanian mainland: 10,940,628 in 2003 (the most recent year for which complete data were available) [23]. The major shortfall of this approach is the unreliability and incompleteness of outpatient data reported through the national health management and information system.

#### Scenario IV – composite estimation

The three scenarios above use a common base to scale up all costs. This has the advantages of simplicity and clarity. However, in reality different bases will be appropriate for different cost components, depending on their key cost drivers. For example, drug costs will be mainly dependent on number of patients, but training costs will be driven more by staff numbers. A composite scenario was therefore calculated as follows, using the most appropriate base for each cost category:

• Project team costs based on 2001 policy change from chloroquine to SP (adjusted for inflation) [14]

• Office equipment costs based on Rufiji District costs

• Development of IEC materials based on actual costs incurred in Rufiji District

• Training of health personnel costs scaled up by multiplying Rufiji District costs per health facility by the total number of registered public and mission health facilities in mainland Tanzania

• Drug purchase and other supplies costs scaled up by multiplying Rufiji District costs per reported malaria outpatient diagnosis by the total national outpatient malaria diagnoses per year reported by the Health Management Information System for all registered public and mission health facilities in mainland Tanzania

• Communication and publicity costs scaled up by multiplying Rufiji District per capita costs by the total population in mainland Tanzania.

From a theoretical perspective we would consider this scenario to be the best estimate of nationwide costs.

In addition, one-way sensitivity analyses were carried out with respect to three key areas: implementation team personnel, increase in health facility utilization, and the use of an alternative less costly ACT. All parameters in the scenarios and sensitivity analyses are presented in Table 2.

### Ethical clearance

This study received ethical approval from the institutional review boards of the Ifakara Health Research and Development Centre, the Tanzanian Medical Research Coordinating Committee, the London School of Hygiene and Tropical Medicine, and the US Centers for Disease Control and Prevention.

## Results

### Costs of Rufiji District implementation

**Figure 2:**
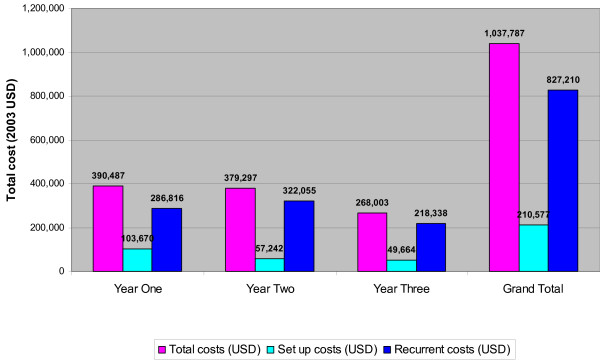
Costs of implementing ACT in Rufiji district (2003 prices).

The costs of deploying ACT in Rufiji District are presented in Table 3 and Figure 2. Results are presented over a period of three years and categorized into set-up and recurrent costs. The total cost for implementing ACT in the district was TZS 1,105,242,773 (1,037,787 USD). Set up costs accounted for 20.3% of total costs over the three years, mainly concentrated in year one, though they continued into years two and three to cover refresher training for health workers and training of council health management teams and primary school teachers. Overall, the most costly set-up items were personnel costs of the project implementation team and consultancy fees, followed by communication and publicity, the costs of developing and producing various IEC materials, and finally office equipment (which included use of a four wheel drive vehicle to supervise implementation). Recurrent costs were fairly constant across the three years, with slightly higher costs in the second year, reflecting an increase in facility utilization and the costs of addressing drug leakage. Overall costs declined over time. The relatively low drug purchase costs in year 3 reflect the use of drugs purchased in the preceding years. The majority of overall costs were related to drug purchase and importation handling charges.

**Table 3:** Total costs of implementing ACT in Rufiji district *(TZS and USD, 2003 prices)*

***COST TYPE***	*Year One*	*Year Two*	*Year Three*	*Grand Total*	%
***Set up costs (Tshs)***					

Project implementation team & consultancy fees	37,443,438	26,852,700	20,248,544	84,544,682	7.6%
Office Equipments	11,152,386	11,152,386	11,152,386	33,457,158	3.0%
Development & Production of IEC materials	22,312,540	11,626,570	4,044,711	37,983,821	3.4%
Training of health personnel	8,161,000	4,616,123	10,523,561	23,300,684	2.1%
Communication and publicity	31,339,600	6,715,355	6,923,259	44,978,214	4.1%
**Total set up costs (TZS)**	**110,408,963**	**60,963,134**	**52,892,460**	**224,264,558**	20.3%
**Set up costs (USD)**	**103,670**	**57,242**	**49,664**	**210,577**	

***Recurrent costs (Tshs)***					

Drug purchase and port handling charges	274,689,940	316,971,141	213,115,713	804,776,794	72.8%
Drug distribution costs	23,227,950	19,693,807	12,568,885	55,490,642	5.0%
Other supplies	7,541,600	6,323,289	6,845,890	20,710,780	1.9%
**Total Recurrent costs (TZS)**	**305,459,490**	**342,988,237**	**232,530,488**	**880,978,215**	79.7%
**Recurrent costs (USD)**	**286,816**	**322,055**	**218,338**	**827,210**	
Grand total (TZS)	415,868,453	403,951,371	285,422,948	1,105,242,773	100%
Total costs (USD)	390,487	379,297	268,003	1,037,787	
***Per capita cost (USD)***	*1.84*	*1.79*	*1.26*	*4.89*	

Newly announced prices for ARLU are lower than what the project was able to negotiate for the purchase of artesunate plus SP. As ARLU has been chosen to be the national first-line antimalarial drug, the results in Rufiji were re-estimated by replacing artesunate plus SP costs with those of ARLU. This led to a reduction of drug purchase costs by 32%, from 755,659 to 529,982 USD. Substituting present-day prices of ARLU for the actual costs incurred purchasing artesunate and SP reduced overall costs of ACT implementation in Rufiji by 22% from 1,037.787 to 807,820 USD over the three year period.

### Scaling up to the national level

Estimated national level costs based on the four scenarios described above are presented in Table 4. Total costs for the first three years of implementation varied from 43.1 to 121.7 million USD, implying a per capita cost of 1.15 to 3.25 USD. Scenario two (scaling up by population) led to the highest cost estimate followed by scenario one (scaling up by number of districts). Scenario three (scaling up by outpatient malaria diagnoses) led to the lowest estimated costs. In Composite Scenario four (considered the best estimate) the costs amounted to 48.3 million USD.

**Table 4:** Summary of costs of three years nationwide implementation of ACTs based on different cost scenarios (2003 USD)

	**Scaled up by:**							
	Number of districts		Population		Number of malaria outpatient diagnoses		Variety of bases (see text)	

***Activities:***	***Scenario I***	***%***	***Scenario II***	***%***	***Scenario III***	***%***	***Scenario IV***	***%***

Project team	82,818	*0.10*	82,818	*0.07*	82,818	*0.19*	82,818	*0.17*
Office Equipments	31,415	*0.04*	31,415	*0.03*	31,415	*0.07*	31,415	*0.07*
IEC development & production	35,666	*0.04*	35,666	*0.03*	35,666	*0.08*	35,666	*0.07*
Training	2,603,551	*3.18*	3,866,486	*3.18*	1,365,999	*3.17*	1,721,766	*3.57*
Drug purchase	63,067,875	*76.92*	93,660,959	*76.97*	33,089,679	*76.79*	33,089,679	*68.55*
Drug distribution costs	8,829,503	*10.77*	13,112,534	*10.78*	4,632,555	*10.75*	4,632,555	*9.60*
Communication & publicity	5,025,735	*6.13*	7,463,627	*6.13*	2,636,841	*6.12*	7,463,627	*15.46*
Other supplies	2,314,162	*2.82*	3,436,720	*2.82*	1,214,166	*2.82*	1,214,166	*2.52*
**Total (USD)**	**81,990,724**	*100*.	**121,690,225**	*100*.	**43,089,138**	*100*.	**48,271,692**	*100*
***Total (TZS)***	**87,320,120,918**		***129,600,089,890***		**45,889,932,230**		**51,409,351,760**	
***Per capita cost (USD)***	***2.19***		***3.25***		***1.15***		***1.29***	

In all four scenarios the single greatest cost was for drug purchase, accounting for between 68.6% and 77.0% of total costs. In scenarios one, two and three, drug purchase costs were followed by drug distribution (around 10.8%). In Composite Scenario four communication and publicity was the second largest cost category (15.5%), followed by drug distribution (9.6%). All other costs were well below 10% of total costs in all scenarios.

### Sensitivity analyses

Based on the best estimate (Composite Scenario four), we undertook a number of one-way sensitivity analyses, covering strengthening the NMCP implementation team, increased facility utilization, and alternative use of a more affordable ACT. Results are summarized in Table 5.

**Table 5:** Sensitivity analysis results for costs of nationwide implementation (using Composite Scenario IV as base case, 2003 USD)

**Activities**	**Base Case (Composite Scenario IV)**	**Strengthening the Implementation team**	**Increased Facility utilization**	**Cheaper ACT**
Implementation team	82,818	290,366	82,818	82,818
Office equipments	31,415	281,487	31,415	31,415
Development of IEC Materials	35,666	35,666	35,666	35,666
Training	1,721,766	1,721,766	1,721,766	1,721,766
Total Drug purchase	33,089,679	33,089,679	49,634,518	9,245,601
Drug Handling & Distribution	4,632,555	4,632,555	6,948,833	1,294,384
Communication & Publicity	7,463,627	7,463,627	7,463,627	7,463,627
Other supplies	1,214,166	1,214,166	1,214,166	1,214,166
**Total (USD)**	**48,271,692**	**48,729,312**	**67,132,809**	**21,089,443**
**Total (TZS)**	**51,409,351,760**	**51,896,717,192**	**71,496,441,307**	**22,460,256,995**
***Percentage change ***		***+0.9%***	***+39.0%***	***-56.0%***

#### Strengthening NMCP team capacity

During the implementation of the 2001 policy change from chloroquine to SP, concerns were raised about the capacity of the NMCP team to implement their programme effectively [14]. The impact on implementation costs was therefore examined of strengthening the NMCP team by establishing five zonal implementation teams. In each zone the teams would comprise a full-time zonal implementation manager, a technical officer, an office assistant, and two health officers, and would each be provided with three months expatriate time to support the establishment of the zonal offices. In addition the national deputy manager post was increased to a full-time job to allow for the additional supervisory activities, and funds were included for supervision of the zonal offices by the national NMCP team, and for zonal office costs. Finally, NMCP salaries were revised based on the new civil servants salary scheme adopted in 2005. Other personnel costs were assumed to remain as recorded in Mulligan et al. This increased the NMCP personnel costs from 82,818 USD to 290,366 USD. The establishment of zonal implementation teams also increased office equipment costs by almost nine times to 281,487 USD. This led to an increase in overall ACT implementation costs in Composite Scenario four model of 0.9%.

#### Increased health facility utilization levels

Household surveys in Rufiji District have shown that after ACT implementation, overall health facility utilization increased from 29% of all care seeking visits for febrile illnesses in 2002, to 43% in 2004 (IMPACT study, unpublished data), which is likely to have reflected awareness that a highly effective antimalarial was available at health facilities. Significant increases were especially noticed among patients aged five years and above. It is, therefore, likely that nationwide provision of a similarly effective antimalarial drug would also increase utilization of public and mission health facilities, assuming that subsidized ACT is not available in the private-for-profit sector. In the sensitivity analysis it was assumed that there would be a 50% increase in national drug purchase costs, which in turn would increase the costs of drug distribution and other supplies. This led to an increase of 39% in overall ACT implementation costs, suggesting that changes in treatment seeking could have a major impact on total programme costs.

#### Use of an alternative ACT

Finally, the use of a less expensive ACT such as artesunate plus amodiaquine was considered. This combination was chosen because it is currently being used in neighbouring Zanzibar as first line drug for treating uncomplicated malaria, with an average cost of 0.85 USD per dose. At this relatively cheaper purchase price, there would be proportionate reduction of drug distribution costs but all other costs would remain constant. This led to a 56% reduction in the overall costs estimated for implementing ACT nationwide.

## Discussion

Given the rise in antimalarial drug resistance and subsequent introduction of new malaria treatment recommendations, it is important to understand the costs of changing antimalarial drug policy in order to facilitate planning by national governments and their donor partners, and as an input into evaluations of the cost-effectiveness and affordability of new drug policies. Studies to date on the national costs of ACT implementation have focused on drug costs [9-11,31] in one case including other case management costs at facility level [13]. No previous studies have fully documented the drug and non-drug costs involved in national policy change to ACT. More importantly, this study is based on data from actual costs incurred during district-wide ACT implementation under real life conditions, in contrast to previous estimates derived from models.

The total cost of three years of ACT implementation in Rufiji District was 1,037,787 USD or USD 4.89 per capita. Depending on the approach used to scale up costs, the nationwide costs of the first three years of ACT implementation were estimated to be between 43.1 and 121.7 million USD, with the best estimate at 48.3 million USD (1.29 USD per capita). This varied between 21.1 and 67.1 million USD in the sensitivity analyses. In all estimates drug costs constituted the majority of total costs. However, non-drug costs such as office equipment, IEC materials, drug distribution, communication, and health worker training were also substantial, accounting for 31.4% of overall ACT implementation costs in the best estimate scenario. Of these non-drug costs, the most expensive were communication and publicity and drug distribution.

In terms of affordability, the implications of ACT implementation for the health budgets of countries like Tanzania are considerable. Tanzania's total health budget (recurrent and development) in 2005 amounted to 254.6 million USD of which 169.3 million USD was allocated for recurrent expenditures, with only 56 million USD remaining for medical supplies [32]. Over 90% of Tanzania's total health development budget is funded through donor support. Between 25 to 45% of the recurrent budget in Tanzania is funded through donor support [33]. Based on Composite Scenario four estimates, the average annual costs of the first three years of national ACT implementation would be equivalent to 9.5% of Tanzania's recurrent health budget, 6.3% of the total health budget (recurrent and development), and 28.7% of total expenditure on medical supplies. The total expenditure on antimalarial drugs in 2005 (USD 1.7 million) would cover only 15.4% of annual ACT drug cost estimates. In view of these scant resources, in 2005 the Global Fund approved a grant of 54.2 million USD to help Tanzania roll out ACTs over a two year period (to cover drug costs and supporting activities) [34]. According to Composite Scenario four, the Global Fund resources would be enough to support ACT policy change for a period of three years. Resources are yet to be identified to sustain the country's policy decision in subsequent years.

These results can be compared with two previous studies in Tanzania. One calculated the non-drug costs of policy change from chloroquine to SP monotherapy; while the other estimated the drug costs of policy change from SP monotherapy to combination therapy (all results have been converted to 2003 USD to facilitate comparison). The first study by Mulligan and colleagues was a post-hoc evaluation of the 2001 policy change from chloroquine to SP, which estimated the nationwide non-drug costs of implementation at 734,441 USD [14], compared with estimates in Composite Scenario IV of non-drug costs which are over 20 times higher at 15,240,144 USD. For example, Mulligan and colleagues reported the average cost per district to be 2,878 USD for training, and 1,483 USD for communication and publicity, compared with the estimates from Rufiji District of 21,879 USD and 42,233 USD respectively. There are a number of possible reasons for these substantial differences. First, it is highly likely that the costs captured were more comprehensive in this study than for the 2001 policy change. The costs in this study were based on thorough data collection at the district level and relied on detailed accounts data and other records established to track costs associated with the delivery of the ACT from the start of the project. In contrast, Mulligan and colleagues estimated the cost of the policy change after the fact and at the macro level. Moreover, the previous study covered the period from 1999 when the Ministry of Health formally cleared the policy change process to August 2002; one year after policy implementation began. The three year period used in this study, therefore, had more comprehensive coverage of costs incurred during post implementation. Secondly, the scope of implementation activities carried out in Rufiji District was broader than those documented for the 2001 policy change, including for example, refresher training for health workers, integration of malaria issues into the primary school curriculum, and intensive communication and publicity activities not matched during the 2001 policy change. One could argue that more intensive activities were warranted to support the introduction of combination therapy compared with a change between two monotherapies, reflecting the more complex dosing regimen, and imperative to ensure good use of these more expensive drugs.

Secondly, a USAID commissioned study by Wolf and Derriennic estimated the projected ACT financing gap for mainland Tanzania in 2007 assuming that ACT was provided through all public and non government facilities, and that 70% of all fever cases were treated in such facilities [11]. The study included drug costs only, evaluating three ACT regimens. It found that the country would need 42.4 million USD per annum to purchase ARLU drugs, 14.6 million USD for artesunate plus amodiaquine, and 19.7 million USD for piperaquine/dihydroartemisinin/trimethoprim (2003 prices). By contrast, the Composite Scenario IV estimate of nationwide ARLU drug costs over three years through the same facilities was 33 million USD, giving an annual average drug cost of 11 million USD, only 24% of the ARLU costs modelled by Wolf and Derriennic. In the sensitivity analysis the estimated drug costs with a more affordable ACT were 9.2 million USD over three years, or 3 million USD per year, compared to the Wolf and Derriennic estimate of 15.5 million USD with artesunate + amodiaquine. There are two major reasons for the substantial differences. One is that Wolf and Derriennic used an older average price per dose for ARLU of 1.57 USD while the current study used a recently negotiated price averaging 1 USD. Wolf and Derriennic estimated their more affordable alternative ACT at 1 USD whereas a more up-to-date average of 0.85 USD per dose was used in this study. In addition, the estimates presented here are based on actual treatment seeking patterns observed in Rufiji District. Although their estimates of fever incidence were based on IMPACT data from study sites including Rufiji District, their assumption that 70% of fever cases will be treated in formal-sector health facilities was not. The 70% figure above is far above the rates observed in Rufiji District either before or after the introduction of ACT (29% and 43% respectively), even though Rufiji District treatment seeking estimates were obtained from household surveys carried out in an area of holoendemic malaria transmission during the rainy season when malaria transmission typically peaks.

There are a number of reasons why nationwide cost estimates based on the experience implementing ACT in Rufiji District could be expected to overestimate costs for the country as a whole. Firstly, though care was taken to maintain 'real life' implementation conditions, the experience in Rufiji District could be considered to represent best practice as it was relatively well-funded, implemented by a highly motivated team and closely monitored. This is likely to have led to a more comprehensive implementation programme beyond what might be expected during routine implementation on a larger scale. Secondly, the ACT used in Rufiji was not co-formulated or co-packaged in one blister pack. This may have necessitated greater resources for additional packaging, IEC and communication than would be required with a co-formulated product such as ARLU. Thirdly, a significant proportion of the Rufiji District population lives in the relatively inaccessible delta area, where the costs of accessing some health facilities and communities are particularly high. Finally, potential savings arising from the use of a more effective drug were not included, such as a decline in severe cases and thus reduced costs of inpatient admissions.

On the other hand, using the actual costs incurred in Rufiji District could equally have led to underestimation of nationwide costs. Firstly, it is likely that the potential for leakage of ACT supplies to private for-profit outlets would be much greater with national level implementation, thus leading to substantial increases in drug and drug distribution costs. Secondly, with national implementation there is the potential to use the mass media for publicity campaigns, which is not feasible on a district level, which in turn could increase implementation costs. Thirdly, Rufiji District is relatively close to Dar es Salaam, facilitating the management of drug distribution and other implementation activities. Moreover, Rufiji District has benefited from the presence of the Tanzania Essential Health Intervention Project (TEHIP) which has helped strengthen the district health system – greater resources might be required to ensure effective management in other settings. Finally, the costs were estimated on an incremental basis; in some settings more fundamental health system strengthening may be required to ensure efficient roll out, such as the recruitment of additional health workers, or improvement of storage facilities to accommodate the shorter life span and bulkier packaging of ACT drugs.

A final limitation is the reliance on malaria outpatient diagnoses data as a base for scaling up all costs in Scenario three and some costs in Scenario four. As in many developing countries, these routine health information system reports are often inaccurate and incomplete. This has a potentially important impact on the cost estimates as the number of malaria diagnoses is the major determinant of Composite Scenario four estimate of national drug requirements, which in turn are the major driver for total ACT deployment costs.

This study's ACT cost estimates were based on the current mode of ACT implementation in Tanzania which involves ACT provision through public and private not-for-profit health facilities only, in most cases on the basis of clinical diagnosis alone. Clinical diagnosis is known to lead to substantial over-treatment with antimalarial drugs [35,36], and the high costs of ACTs and concern about excessive drug pressure have led to calls for improvements in malaria diagnosis by extending the use of microscopy and rapid diagnostic tests (RDTs). The impact of increased reliance on parasite-based diagnosis on implementation costs is beyond the scope of this paper but would be affected by malaria parasite prevalence, diagnostic accuracy, the cost of the test and prescribing behaviour of clinicians particularly when faced with a negative test result [35,37]. In addition, there have been calls for ACT coverage to be expanded through the use of private retailers such as drug shops, and/or village health workers [38,39]. This would require a substantial increase in non-drug resources to cover additional activities such as training and communication, and if successful in increasing coverage, could also lead to major increase in total drug costs.

## Conclusion

This study has improved the evidence base for governments on the incremental financial costs of ACT implementation. The estimates were based on a detailed assessment of costs incurred during district-wide implementation in a typical rural district, and the extrapolation of these data to estimate nationwide costs. They included both drug and non-drug costs, such as IEC materials, training of health personnel, communication, drug distribution, other supplies and the project implementation team.

The costs are substantial, with a best estimate of 48.2 million USD (1.29 USD per capita) over the first three years. The total overall costs varied between 21 and 67.1 million USD in the sensitivity analysis. The study highlights three key messages. Firstly, drug costs constituted the majority of total costs, but non-drug costs were also considerable, accounting for 31.4% of overall costs in Composite Scenario IV estimate. Secondly, rolling out ACTs would lead to a more than 6-fold increase in the national budget for antimalarial drugs. Thirdly, average annual implementation costs over the first three years would be equivalent to 9.5% of the total health sector recurrent budget. It is thus clear that substantial external resources are required to facilitate implementation of ACT, particularly in the early years of implementation, but also to ensure the sustainability of effective provision of malaria medicines.

## Authors' contributions

Njau & Goodman collected, analyzed the data and drafted the paper. Njau, Goodman, Mulligan, Kachur, and Abdulla, designed the study while Munkondya and Mchomvu assisted with the data collection process. Mills and Bloland provided overall supervision. All authors contributed to the final manuscript.

## IMPACT-Tz Project and Funding

The Interdisciplinary Monitoring Project for Antimalarial Combination Therapy in Tanzania (IMPACT-Tz) is a multiyear implementation research evaluation project that rests on a collaborative platform comprising the US Centers for Disease Control and Prevention (CDC), Ifakara Health Research and Development Centre, the National Institute for Medical Research, Muhimbili University College of Health Sciences, the London School of Hygiene and Tropical Medicine (UK) and the Tanzanian MoH, including its National Malaria Control Programme, the Tanzania Essential Health Interventions Project, and the Council Health Management Teams of Rufiji, Morogoro, Mvomeru, Kilombero and Ulanga Districts. IMPACT-Tz is primarily supported by funding from CDC, the United States Agency for International Development and Wellcome Trust.

## Disclaimer and Competing interests

The findings and conclusions presented are those of the authors and do not necessarily represent those of the United States Public Health Services (USPHS) nor the Centers for Disease control and Prevention (CDC). Trade names are used for identification purposes only and do not imply endorsement by USPHS nor CDC.

All authors of this article declare to have no financial or other interest with any organization or entity that may influence or be considered as constituting a real, potential or apparent conflict of interest to bias whatever findings presented in this study.
